# Drug Efflux Transporters Are Overexpressed in Short-Term Tamoxifen-Induced MCF7 Breast Cancer Cells

**DOI:** 10.1155/2016/6702424

**Published:** 2016-02-14

**Authors:** Desak Gede Budi Krisnamurti, Melva Louisa, Erlia Anggraeni, Septelia Inawati Wanandi

**Affiliations:** ^1^Department of Medical Pharmacy, Faculty of Medicine, University of Indonesia, Jakarta 10430, Indonesia; ^2^Department of Pharmacology and Therapeutics, Faculty of Medicine, University of Indonesia, Jakarta 10430, Indonesia; ^3^Master Program in Biomedicine, Faculty of Medicine, University of Indonesia, Jakarta 10430, Indonesia; ^4^Department of Biochemistry and Molecular Biology, Faculty of Medicine, University of Indonesia, Jakarta 10430, Indonesia

## Abstract

Tamoxifen is the first line drug used in the treatment of estrogen receptor-positive (ER+) breast cancer. The development of multidrug resistance (MDR) to tamoxifen remains a major challenge in the treatment of cancer. One of the mechanisms related to MDR is decrease of drug influx via overexpression of drug efflux transporters such as P-glycoprotein (P-gp/MDR1), multidrug resistance associated protein (MRP), or BCRP (breast cancer resistance protein). We aimed to investigate whether the sensitivity of tamoxifen to the cells is maintained through the short period and whether the expressions of several drug efflux transporters have been upregulated. We exposed MCF7 breast cancer cells with tamoxifen 1 *μ*M for 10 passages (MCF7 (T)). The result showed that MCF7 began to lose their sensitivity to tamoxifen from the second passage. MCF7 (T) also showed a significant increase in all transporters examined compared with MCF7 parent cells. The result also showed a significant increase of CC50 in MCF7 (T) compared to that in MCF7 (97.54 *μ*M and 3.04 *μ*M, resp.). In conclusion, we suggest that the expression of several drug efflux transporters such as P-glycoprotein, MRP2, and BCRP might be used and further studied as a marker in the development of tamoxifen resistance.

## 1. Introduction

Tamoxifen has been used as first line treatment for estrogen receptor alpha- (ER*α*-) positive breast tumors in women for many years [1–3]. However, resistance to tamoxifen occurs in many patients, although ER*α* expression is maintained in most tumors that acquire resistance [4].

Many factors contribute to tamoxifen-acquired resistance, involving a number of profound changes in the expression of genes, including multidrug resistance (MDR) phenomenon [5–7]. Several mechanisms have been proposed to explain MDR, one of which is decreased intracellular drug accumulation which resulted from a decrease of drug influx via overexpression of drug efflux transporters such as P-glycoprotein (P-gp/MDR1), multidrug resistance associated protein (MRP), or BCRP (breast cancer resistance protein) [8, 9]. Studies have shown that the overexpression of multidrug resistance (MDR) played an important role in the development of cancer resistance to tamoxifen [8, 10, 11].

Tamoxifen-acquired resistance cell lines have been obtained by researchers by adding tamoxifen to cell lines for months and years [10–12]. Although these cell-based studies of MDR have been an important source of understanding about the mechanism of resistance, no data showed whether overexpression of drug efflux transporters, which leads to resistance to tamoxifen, occurs in a much shorter regimen. In this study, we used a short-term tamoxifen treatment to MCF7 cells and determined whether the sensitivity of drugs to the cells is maintained through the short period and whether the expression of several drug efflux transporters has been upregulated.

## 2. Materials and Methods

### 2.1. Materials

MCF7 cell line for breast cancer was a kind gift from the Laboratory of the Agency for the Assessment and Application Technology (BPPT), Serpong, Indonesia. Tamoxifen and DMSO were purchased from Sigma-Aldrich (Singapore). Dulbecco Minimal Essential Medium (DMEM), Fetal Bovine Serum (FBS), Penicillin/Streptomycin, Gentamicin, Fungizone, Dulbecco Phosphate Buffer Solution (D-PBS), and Triple Express were obtained from Gibco, Ltd. (Singapore). Tripure Isolation Reagents were from Roche Diagnostics (Singapore), primers were from 1st BASE Ltd., Singapore, and qRT-PCR kit used was KAPA SYBR FAST One-Step qRT-PCR Kit Universal from KAPA Biosystem, USA. MTS assay kit was obtained from Promega, USA.

### 2.2. Cell Culture

MCF7 cells were cultured in DMEM supplemented with 10% heat-inactivated fetal bovine serum, 2 mM L-glutamine, 100 IU/mL penicillin, 100 *μ*g/mL streptomycin, and 1% Fungizone. Medium was routinely changed every day. The cells were subcultured when reaching 80–90% confluence.

### 2.3. Tamoxifen-Induced MCF7 Cells

MCF7 cells were grown in a medium containing tamoxifen 1 *μ*M, continuously up to 10 passages (44 days). When reaching confluence, cells were subcultured and counted using trypan blue exclusion method. For the purpose of cell viability counting, we normalized the data to DMSO, as control, and show the data as % viability over control. Cells from passage 1 (day 5) and passage 10 (day 44) were subjected to RNA isolation and qRT-PCR for drug efflux transporters (P-glycoprotein, MRP2, and BCRP).

### 2.4. Cell Morphology

MCF7 and MCF7 (T) cells morphology were photographed under confocal microscope (Olympus Fluoview FV1200 Confocal Laser Scanning Microscope, Olympus, Japan). Photo observation is done using gray scale, Pseudo 3D DIC by Transmitted Nomarski System.

### 2.5. RNA Isolation

Total RNA was isolated using Tripure Isolation Reagents (Roche) according to the manufacturer's protocol. Quantity and purity of RNA were determined by measuring 260/280 absorbance using NanoDrop spectrophotometer. RNA obtained then was subjected to quantitative real-time reverse transcription polymerase chain reactions (qRT-PCR).

### 2.6. qRT-PCR

mRNA expressions of the following drug transporter were quantified: P-glycoprotein, MRP2 (multidrug resistance protein-2), and BCRP (breast cancer resistance protein). Quantitative real-time reverse transcription polymerase chain reaction (qRT-PCR) was performed using KAPA SYBR FAST One-Step qRT-PCR Kit on Universal Biorad Chromo 4 Real-Time PCR Detection System. *β*2-microglobulin was used as reference gene. The sequences of the primers were *β*2mg F: CCAGCAGAGAATGGAAAGTC; *β*2mg R: CATGTCTCGATCCCACTTAAC. Primers used for the determination of drug efflux transporters were described previously [13]: P-glycoprotein, P-gp F: CCCATCATTGCAATAGCAGG; P-gp R: TGTTCAAACTTCTGCTCCTGA; MRP2, MRP2 F: ACAGAGGCTGGTGGCAACC; MRP2 R: ACCATTACCTTGTCACTGTCCATGA; BCRP, BCRP F: AGATGGGTTTCCAAGCGTTCAT; BCRP R: CCAGTCCCAGTACGACTGTGACA. Primers used to determine the mRNA expressions of Caspase-3 and Caspase-9 were described previously by Iwao et al. [14] with sequence as follows: Cas-3 F: TTCAGAGGGGATCGTTGTAGAAGTC; Cas-3 R: CAAGCTTGTCGGCATACTGTTTCAG; Cas-9 F: ATGGACGAAGCGGATCGGCGGCTCC; Cas-9 R: GCACCACTGGGGGTAAGGTTTTCTAG. Primers used to determine the mRNA expression of progesterone receptor were used previously by Shanker et al. [15]: PR F: GGCGGATCCGTCAAGTGGTCTAAATCATTG; PR R: GGCGAATTCCTGGGTTTGACTTCGTAGCCC. Relative changes in mRNA transporter expression levels were calculated using Livak method [16].

### 2.7. MTS Assay (Cell Proliferation Assay)

Cytotoxicity concentration of tamoxifen to MCF7 cells before and after 44-day treatment of tamoxifen was determined using MTS assay (Promega). Cells were plated at a density of 2000 cells per well in 96-well plates. At 70–80% confluence, cells were incubated with tamoxifen for 24 h at 37°C. After 24 h drug treatment, 20 *μ*L of MTS solution was then added into each well and incubated for 2 h before reading at a wavelength of 490 nm. CC50 values were calculated from linear regression equation of dose-response curves.

### 2.8. Statistical Analysis

Data were presented in the form of means ± standard deviation (SD). Graphs were created using GraphPad Prism software 6 (GraphPad, USA). Statistical significance was calculated using *t*-test or ANOVA One-Way followed by post hoc test, with *p* < 0.05 considered as significant.

## 3. Results

**Figure 1:**
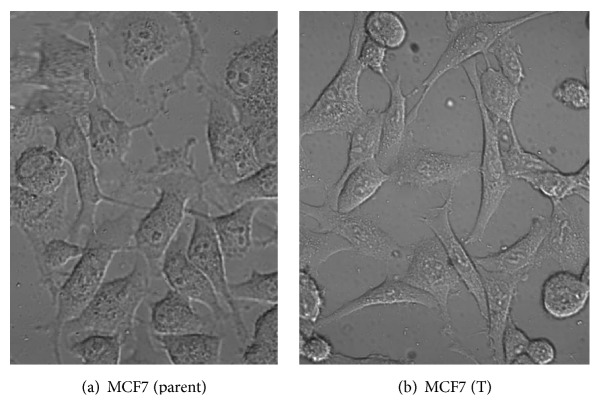
(a) Cell morphology of MCF7 cells (parent). (b) MCF7 (T) cells treated with tamoxifen 1 *μ*M for 10 passages (44 days). Photographed under confocal microscope.

Cell morphology of MCF7 cells treated with tamoxifen continuously is shown on Figure 1.

**Figure 2:**
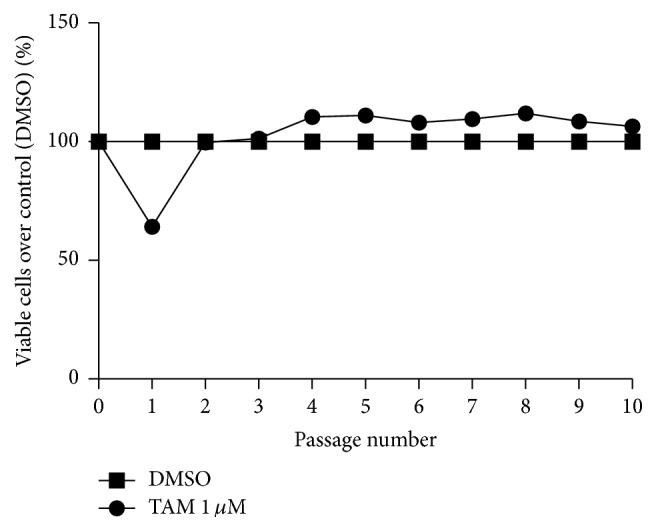
Percentage of viable cells over control (DMSO) after treatment with tamoxifen 1 *μ*M or DMSO.

Our result showed that cancer cells maintained their sensitivity towards tamoxifen only in the first passage. Cells began to lose their sensitivity to drug from the second passage (or about 9 days of tamoxifen treatment). Afterwards, MCF7 treated with tamoxifen had stable overgrowth compared with MCF7 cells treated with DMSO only (Figure 2).

**Figure 3:**
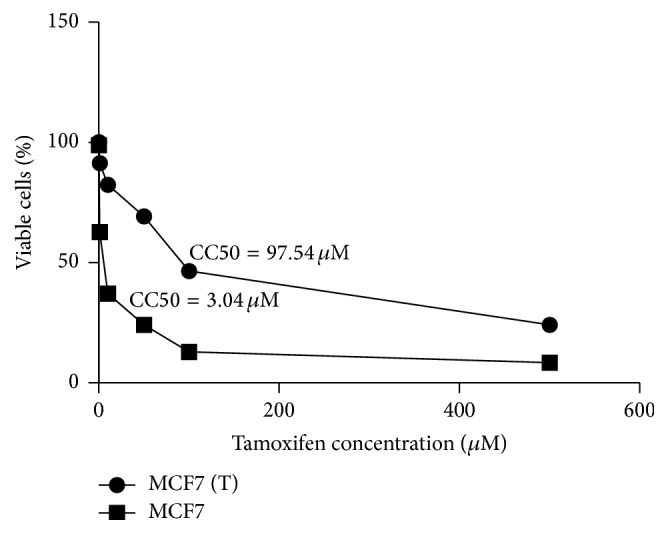
Cytotoxicity concentrations 50 (CC50) of tamoxifen in MCF7 or MCF7 (T) cells.

After 10 passages (44 days) of treatment, we checked the cytotoxicity concentrations of tamoxifen in MCF7 and MCF7 (T). We found a significant increase of CC50 in MCF7 (T) compared to that in MCF7 (97.54 *μ*M and 3.04 *μ*M, resp.) (Figure 3).

**Figure 4:**
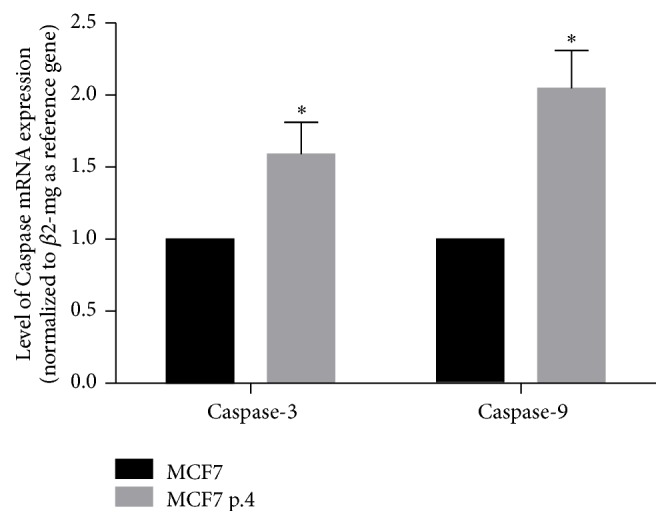
Level of Caspase-3 and Caspase-9 mRNA expressions after treatment with tamoxifen 1 *μ*M at passage 4. Results were shown as mean ± SD (*N* = 4). (*∗*) Significant difference versus MCF7 parent cells at *p* < 0.05.

In order to show whether the apoptosis process is still active in MCF7 cells treated continuously with tamoxifen, we measured Caspase-3 and Caspase-9 mRNA expressions at passage 4 after drug treatment (Figure 4). We found that Caspase-3 and Caspase-9 expressions were significantly increased compared with parent cells, which proved that apoptosis process, which were still in place.

**Figure 5:**
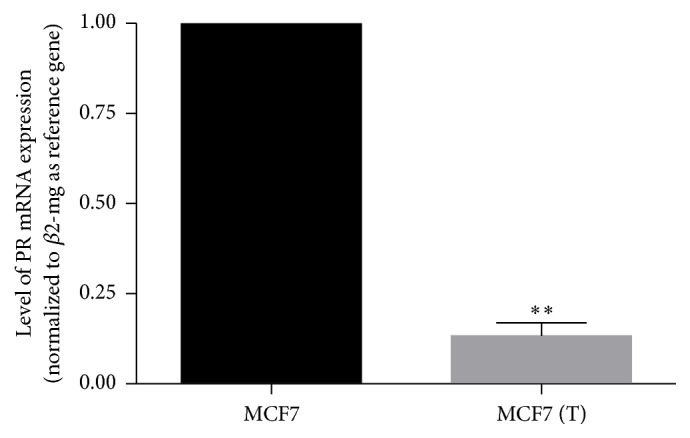
Level of progesterone receptor expressions after 44 days of treatment with tamoxifen 1 *μ*M MCF7 (T). Results were shown as mean ± SD (*N* = 4). (*∗∗*) Significant difference versus MCF7 parent cells at *p* < 0.001.

We found that PR receptor expression was significantly downregulated in MCF7 (T) compared to MCF7 parent cells as shown in Figure 5.

**Figure 6:**
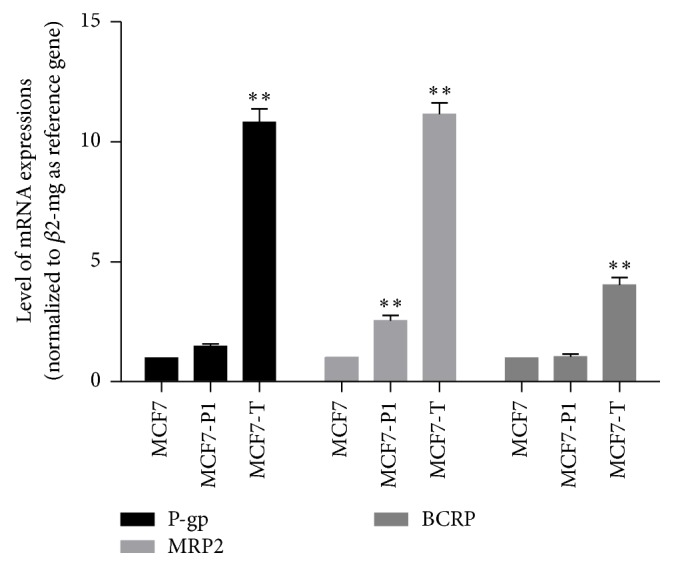
Level of mRNA expressions of P-glycoprotein, MRP2, and BCRP after 5 days of treatment (MCF7-P1) or 44 days of treatment (MCF7-T) with tamoxifen 1 *μ*M. Results were shown as mean ± SD (*N* = 4); (*∗∗*) Significant difference versus MCF7 parent cells at *p* < 0.001.

We measure the expressions of P-gp, MRP2, and BCRP in MCF7 parent cells, MCF7-P1 (MCF1 passage 1), and MCF7 (T). The result showed that the expressions of P-gp, MRP2, and BCRP had been elevated from the first passage. MCF7 (T) showed a significant increase in all transporters examined compared with MCF7 parent cells (Figure 6).

## 4. Discussion

Tamoxifen currently is still the mainstay of endocrine therapies for ER*α*-positive breast tumors [1]. Unfortunately, majority of patients treated with tamoxifen eventually develop resistance, leading to disease progression and death [5]. Tamoxifen-resistant breast cancer cells often overexpress drug efflux transporter, which lower the effective drug concentration in a cell by pumping out tamoxifen out of the cells [6].

Acquired resistance to anticancer mostly occurred after long-term exposure to drugs [17]. To our knowledge, this is the first to describe the expressions of several drug transporters after short period of tamoxifen treatment. Previous studies had described the development of resistance of breast cancer cells to tamoxifen by exposing the drug for years [8, 11, 18, 19]. In this study, we use 1 *μ*M tamoxifen as a treatment in breast cancer cells, as also used by Motahari and Lykkesfeldt [12, 19]. Fewer studies had used lower dose of tamoxifen compared to this study [20]. Our own preliminary result (data not shown) using tamoxifen 0.1 *μ*M and 0.25 *μ*M had resulted in about 90% viability over control during the first passage. Therefore, we thought tamoxifen in lower dosages had little effect on cell viability and thus would result differently in selection of cells to induce resistance.

After 10 passages of tamoxifen treatment, photographs using confocal microscope indicate that there might be slight changes in cell morphology. We found more mesenchymal-like cells in MCF7 (T) compared to MCF7 parents cells which showed more cobblestone-like cells. Other studies had shown that epithelial-mesenchymal transition process played significant roles in the development of tamoxifen resistance [21–23]. In this study, we did not confirm the markers of EMT, as we mainly aimed to determine drug efflux expressions in tamoxifen-resistant cells.

Our result showed that downstream regulation of ER had occurred, as confirmed with downregulation of progesterone receptor. This is in accordance with previous results that tamoxifen resistance is accompanied with the reduced expressions of both ER and PR. In his study, Johnston et al. had also used ER/PR ratio as prognostic markers to tamoxifen resistance [24].

In this study, we showed that reduced sensitivity of cancer cells to tamoxifen developed very early, followed by stable growth up to 10 passages. Tamoxifen had failed to suppress cancer cell growth as early as second passage. We found that apoptosis process was still ongoing in passage 4 as shown by increased expressions of Caspase-3 and Caspase-9. Previous studies using MCF7 and MDA-MB-231 had also shown that tamoxifen, apart from its actions on ER, is able to induce apoptosis process trough cleavage of retinoblastoma (Rb) protein and activation of Caspase-3 [25].

We evaluate the role of drug efflux transporter inhibitors in the parent cells, in first passage (which still showed anticancer activity), and at MCF7 (T). Our result suggests that tamoxifen dramatically increased mRNA expressions of P-glycoprotein, MRP2, and BCRP. The expressions of the three drug transporters mRNA had even began to increase from first passage. Mechanism of modulation of P-glycoprotein and BCRP expressions is reported by Chen and Nie, which suggests that upregulation of mRNA P-glycoprotein and BCRP by tamoxifen occurs through the activation of pregnane X receptor, master regulator of MDR in cancers [26]. Another study by Nagaoka reported that tamoxifen activates CYP3A4 and MDR1/P-glycoprotein genes through steroid and xenobiotic receptor (SXR), a member of nuclear hormone receptors, which may affect tamoxifen metabolism and transport in breast cancer cells [27].

Tamoxifen strongly affects MRP2 expressions in MCF7 cells. Our result is in accordance with Choi et al. who found that tamoxifen-resistant MCF7 cells expressed a very higher level of MRP2 than control MCF7 cells [10]. Choi also found that pregnane X receptor (PXR) was persistently activated in tamoxifen-resistant MCF7 cells [10]. As PXR activates both P-glycoprotein and MRP2, presumably PXR have significant contribution to the development of tamoxifen-resistant breast cancer cells [28].

After 10 passages of treatment with tamoxifen, we found there is 32-fold increase in CC50 of MCF7 (T) compared with that in parent cells. This is a very large magnitude of increase considering a short period of tamoxifen treatment.

Our result suggests that resistance of breast cancer cells to tamoxifen might develop very early, only after a short period of treatment. We suggest that the expression of several drug efflux transporters such as P-glycoprotein, MRP2, and BCRP might be used and further studied as a marker in the development of tamoxifen resistance.
